# Semantic Search of FDA Guidance Documents Using Generative AI

**DOI:** 10.1007/s43441-025-00798-8

**Published:** 2025-06-14

**Authors:** Scott Proestel, Linda J. B. Jeng, Christopher Smith, Matthew Deady, Omar Amer, Mohamed Ahmed, Sarah Rodgers

**Affiliations:** 1 Division of Biomedical Informatics, Research, and Biomarker Development, Office of Drug Evaluation Sciences, Office of New Drugs, Center for Drug Evaluation and Research, FDA, 10903 New Hampshire Ave., Silver Spring, MD 20993 USA; 2 International Business Machines (IBM), Washington D.C, USA

**Keywords:** FDA guidance, Generative AI, Large language model, Document search

## Abstract

**Introduction:**

Generative artificial intelligence (AI) has the potential to transform and accelerate how information is accessed during the regulation of human drug and biologic products.

**Objectives:**

Determine whether a generative AI-supported application with retrieval-augmented generation (RAG) architecture can be used to correctly answer questions about the information contained in FDA guidance documents.

**Methods:**

Five large language models (LLMs): Flan-UL2, GPT-3.5 Turbo, GPT-4 Turbo, Granite, and Llama 2, were evaluated in conjunction with the RAG application Golden Retriever to assess their ability to answer questions about the information contained in clinically oriented FDA guidance documents. Models were configured to precise mode with a low temperature parameter setting to generate precise, non-creative answers, ensuring reliable clinical regulatory review guidance for users.

**Results:**

During preliminary testing, GPT-4 Turbo was the highest performing LLM. It was therefore selected for additional evaluation where it generated a correct response with additional helpful information 33.9% of the time, a correct response 35.7% of the time, a response with some of the required correct information 17.0% of the time, and a response with any incorrect information 13.4% of the time. The RAG application was able to cite the correct source document 89.2% of the time.

**Conclusion:**

The ability of the generative AI application to identify the correct guidance document and answer questions could significantly reduce the time in finding the correct answer for questions about FDA guidance documents. However, as the information in FDA guidance documents may be relied on by sponsors and FDA staff to guide important drug development decisions, the use of incorrect information could have a significantly negative impact on the drug development process. Based on our results, the correct citation documents can be used to reduce the time in finding the correct document that contains the information, but further research into the refinement of generative AI will likely be required before this tool can be relied on to answer questions about information contained in FDA guidance documents. Rephrasing questions by including additional context information, reconfiguring the embedding and chunking parameters, and other prompt engineering techniques may improve the rate of fully correct and complete responses.

**Supplementary Information:**

The online version contains supplementary material available at 10.1007/s43441-025-00798-8.

## Introduction

The U.S. Food and Drug Administration (FDA) has published over 2700 guidance documents, which are used to publicly disseminate FDA’s current thinking on topics related to pharmaceutical development. Existing technologies allow the use of predetermined filters or keyword searches to identify relevant guidance documents. However, this limits the selection of documents to those with exact-match keywords in the title, and there is currently no simple way to search for information contained within the documents. Alternative techniques, such as semantic search, can improve the accuracy of document identification as they assess the intent and contextual meaning of a question. Pairing semantic search with generative AI could allow users to search for both the documents that contain the information request and answer the question posed using information contained within the guidance documents. In this scenario, a user would enter a question into the tool and receive a response based on the information contained in the guidance document, as well as a listing of the guidance documents used to formulate the response.

Generative AI has emerged as a significant advance in recent years due to the development of large language models which demonstrate high performance on a wide range of tasks around understanding and responding to human language [1]. However, the use of these powerful AIs presents risks in the medical [2], pharmacovigilance [3], and public sector domains [4]. To prevent these risks, frameworks have been set up for trustworthy and ethical AI which focus on validation of outcomes. However, validating outcomes is often more difficult for LLMs given the wide range of tasks they can perform. LLMs are assessed on a wide variety of tasks using benchmarks such as GLUE [5, 6] and BIG-bench [7], but this is often a poor test for their performance on tasks specific to the medical domain, including understanding highly specific domain terminology, sorting through a high volume and complexity of information, and doing so at a high level of accuracy given the significant implications for public health and safety. Task-specific validation can be used for real-world applications, such as ours, to confirm the trustworthiness of AI outcomes [8]. The goal of this study was to perform such a validation to assess the degree to which generative AI can be used to successfully perform this task.

## Materials and Methods

FDA draft and final guidance documents [9] in portable document format (PDF) were used as the primary data source for this study. As the focus of this work was to assess the ability of generative AI to help FDA clinical reviewers find information in guidance documents, the documents were limited to those considered clinically relevant by a team of subject matter experts (SMEs). The following guidance topics were determined to not be typically applicable to clinical reviewers and were therefore excluded: Chemistry, Manufacturing, and Controls; Current Good Manufacturing Practice; Drug Competition Action Plan; Export; Human Food Safety; ICH – Quality; Import; Investigational Device Exemption; Pharmaceutical Quality; Pharmacology/Toxicology; and User fees. Excluding these topics resulted in 711 guidance documents being included in the data repository. Metadata were collected for each guidance document which consisted of guidance title, issue date, FDA organization, topic, guidance status, and product type.

Given limited funding and time, the SMEs were not able to generate questions, expected answers, and evaluate results for each of the 711 documents. Instead, they selected a subset that were broadly applicable across clinical disciplines (i.e., not specific to one therapeutic area) which resulted in the identification of 112 documents that were then used to test the LLMs. Documents with either general or limited applicability were filtered out, including those that were disease-specific, focused on specific populations, or were less relevant to clinical reviewers. The 112 selected documents presented the best test of the RAG application [10] because they were the most likely to be used as sources for clinical reviewers’ questions.

The first stage of this project was to test various LLMs and determine which performed best with respect to answering questions related to information contained in FDA guidance documents. The following LLMs [11] were tested:

1. Flan-UL2 [12].
2. GPT-3.5 Turbo [13].
3. GPT-4 Turbo [14].
4. Granite [15].
5. Llama 2 [16].

These LLMs were chosen because they were the best performing LLMs available at the start of the study in September 2023.

A two-stage approach was used to first evaluate performance across all the LLMs, followed by more extensive testing on the highest performing model. To evaluate the five LLMs, 10 of the 112 guidance documents were randomly selected. An analyst examined the documents to create question and expected answer pairings for each of the 10 guidance documents. A variety of question types were included to assess whether one type of question performed better than the others. Question types included yes-or-no response, list, open-ended, prompted, and prompt where the answer was found in a table. To evaluate LLM performance, the analyst entered a question into IBM Golden Retriever, a retrieval-augmented generation (RAG) application [17], which in turn generated a response accompanied with citations, as seen in Fig. s1. RAG is an architecture that enhances LLMs by retrieving relevant contents from an external source for LLMs to produce context-aware responses. This approach improves factual accuracy, reduces the frequency of hallucinations, and makes generative AI outputs more reliable by grounding them in real-world information. IBM Golden Retriever (which has no connection with a similarly named Golden Retriever RAG tool [18]) presents notable advantages over alternative Enterprise RAG solutions, including superior retrieval relevancy by integrating multi-level semantic text embeddings, scalability to millions of documents as the knowledge base, flexible deployment options, and compatibility with any LLM model.

To consistently evaluate the performance of the LLMs across the guidance documents, a scoring rubric was established (Table 1) that would categorize every answer from the RAG tool into one of four categories. A team of three clinical SMEs independently reviewed each response, and, based on the closeness to the reference answer, assigned the response to one of the four categories using the scoring criteria. Inter-rater reliability (IRR) between the three clinician SMEs was calculated using Fleiss’ kappa [19]. The independent scores were discussed among the clinical SMEs and a final consensus score was determined for each response. The proportion, along with a 95% confidence interval (CI) [20], of the four scoring categories for these final scores out of all questions, were then calculated for both stages of the study. The evaluation and scoring system described above was developed to best understand how well the Golden Retriever application would provide useful and/or completely correct answers to complicated, domain-specific questions that would be asked by FDA clinical reviewers.

**Table 1:** Scoring criteria

Score	Name	Criteria
1	Contains incorrect information or no relevant information	The generated text provides none of the requested information or the response contained at least some incorrect information
2	Contains some correct information	The generated text answered at least some of the key aspects of the question and no incorrect information was provided or additional information beyond the expected answer was provided and was considered not helpful or contained poor grammar
3	Correct response	The generated text fully addressed all key aspects of the question, and no incorrect information was contained in the response
4	Correct response with additional helpful information	The generated text fully addressed all key aspects of the question, no incorrect information was provided, and additional information beyond the expected answer was provided and considered helpful

**Fig. 1:**
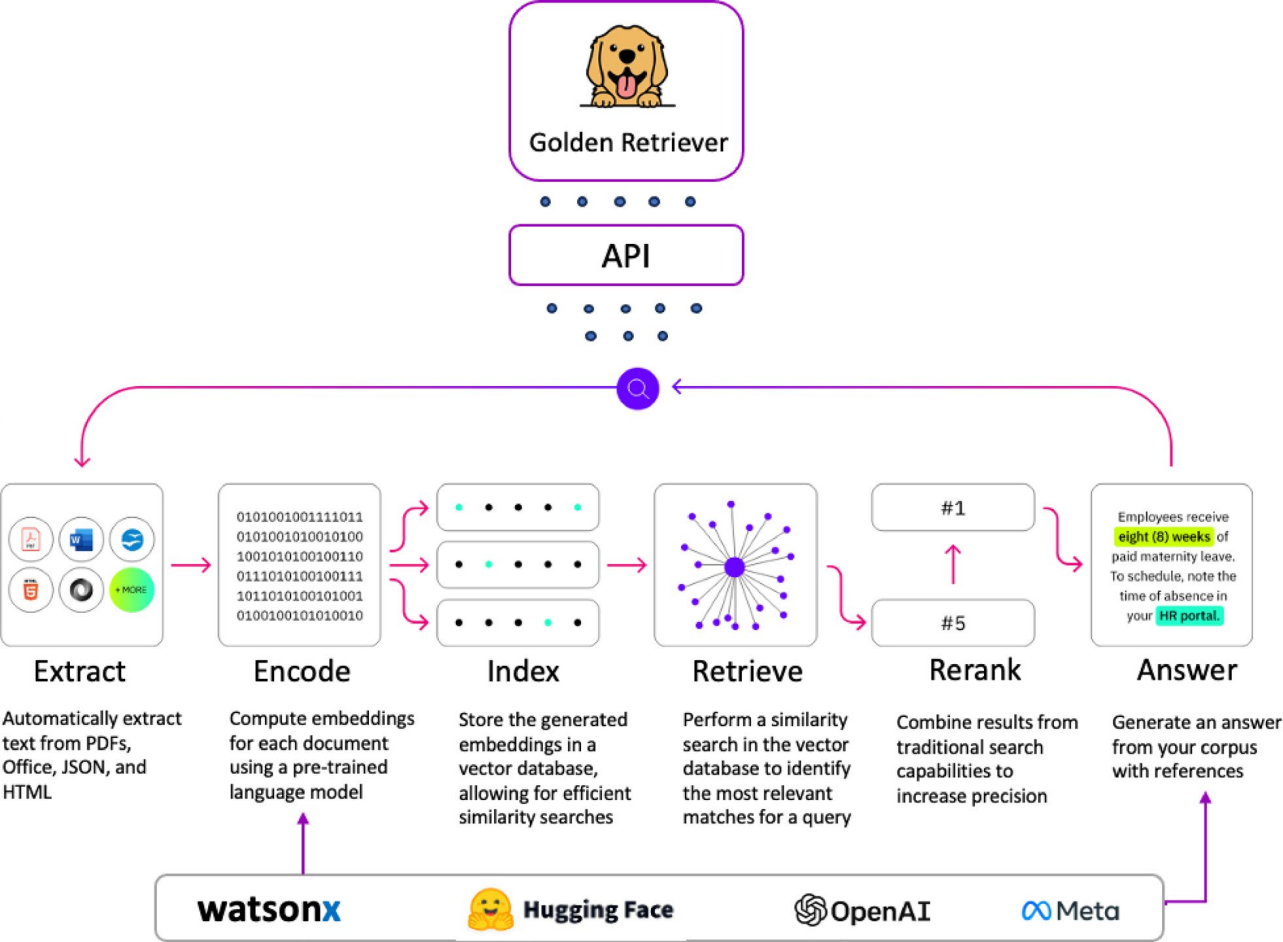
Golden Retriever architecture

After the five LLMs were assessed, we assessed the performance of the embedding model to identify citation documents and the ability of the highest scoring LLM to answer questions using the remaining 112 selected guidance documents. Similar to the testing of all five LLMs, an analyst created test questions and expected answers for each document and used the Golden Retriever application to produce a response accompanied with up to three document citations. Table s1 provides the number of questions created for each question type. The same team of clinical SMEs reviewed and scored the responses using the scoring criteria in Table 1. For both tests, the documents were identified, and questions were answered by Golden Retriever. Golden Retriever answered the questions by reviewing the 711 documents applicable to clinical reviewers which were ingested into the tool using the process described in Fig. 1. Golden Retriever is an asset layer that sits on top of an LLM platform and serves as an accelerator to ingest, index, and query across many documents. The tool extracts the text from PDF documents and computes embeddings for each document using the msmarco-bert-base-dot-v5 embedding model [21]. These embeddings represent the similarity of documents and are stored into a vector database. When the user inputs a question, the most relevant documents for that query are identified using the vector database. The relevant documents are then passed as context along with any pre-defined prompt information that would apply to all questions to the LLM to generate a response, and the Golden Retriever application displays the citation within the output. Often, different prompts are tested to assess the best performing language to retrieve the necessary information in a process known as prompt engineering. Given constraints on time and materials for this study, we only developed a single prompt to be used for all studies which is found in the Supplemental Materials section.

The Golden Retriever application allows for the user to select various hyperparameters to tune the desired output. This includes the following hyperparameters:

- *Context Window* The number of words that the LLM evaluates before and after the selected text when generating the output.
- *Mode* The predictability of the output, also known as temperature. Options include precise, balanced, and creative. Precise makes the model deterministic whereas balanced makes it slightly more imaginative. Creative produces a more creative and ‘outside-the-box’ output.
- *Model* A listing of the available LLMs.
- *Number of Results in Context* The number of documents that the result can be found in.

For this project, the mode was set to precise, ensuring reliable clinical regulatory review guidance for users. The context window was set to two words to conserve space, given the limited context capacity of typical LLMs in 2023. The number of results in context was set to three to balance information richness while preventing LLMs from being overwhelmed by noise and diluted information. Future experiments on context window and number of results in context can be conducted to determine the optimal values for this use case.

## Results

**Fig. 2:**
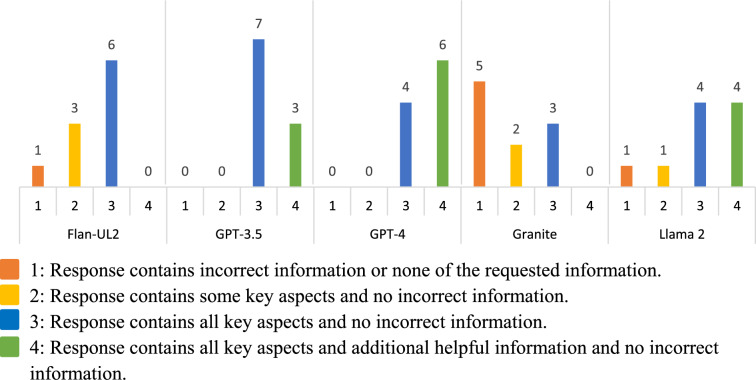
Comparison of LLM performance in RAG tool on question answering task on guidance documents

For our initial LLM question answering capability assessment on the 10 questions for the randomly selected documents, GPT-4 Turbo performed best, followed by GPT-3.5 Turbo, Llama 2, Flan-UL2, and Granite. These results are presented in Fig. 2 and in fraction form in Table 2. The answers generated with GPT-4 Turbo all fully addressed all key aspects of the question and contained no incorrect information. These answers received a median score of 4, indicating that the majority of the text generated by this LLM (answers to 6 of the 10 questions) also provided helpful additional information beyond the expected answer. GPT-3.5 Turbo also performed well with all responses fully addressing all key aspects of the question and containing no incorrect information. However, when comparing these two models, GPT-4 Turbo typically provided additional helpful information that wasn’t expected, whereas GPT-3.5 Turbo typically provided only the expected answer. The majority of responses generated by Llama 2 and Flan-UL2 were correct; however, some responses contained incorrect information or were partial responses. Half of the responses generated by Granite provided either none of the requested information or incorrect information.

**Table 2:** LLM assessment results

LLM assessed	Scored value	N	% [95% CI]
Flan	1	1	10 [1.8, 40.4]
	2	3	30 [10.8, 60.3]
	3	6	60 [31.3, 83.2]
	4	0	0 [0.0, 28.0]
GPT35	1	0	0 [0.0, 28.0]
	2	0	0 [0.0, 28.0]
	3	7	70 [40.0, 89.0]
	4	3	30 [10.8, 60.3]
GPT4	1	0	0 [0.0, 28.0]
	2	0	0 [0.0, 28.0]
	3	4	40 [17.0, 69.0]
	4	6	60 [31.3, 83.2]
Granite	1	5	50 [24.0, 76.0]
	2	2	20 [5.7, 51.0]
	3	3	30 [10.8, 60.3]
	4	0	0 [0.0, 28.0]
Llama 2	1	1	10 [1.8, 40.4]
	2	1	10 [1.8, 40.4]
	3	4	40 [17.0, 69.0]
	4	4	40 [17.0, 69.0]

**Fig. 3:**
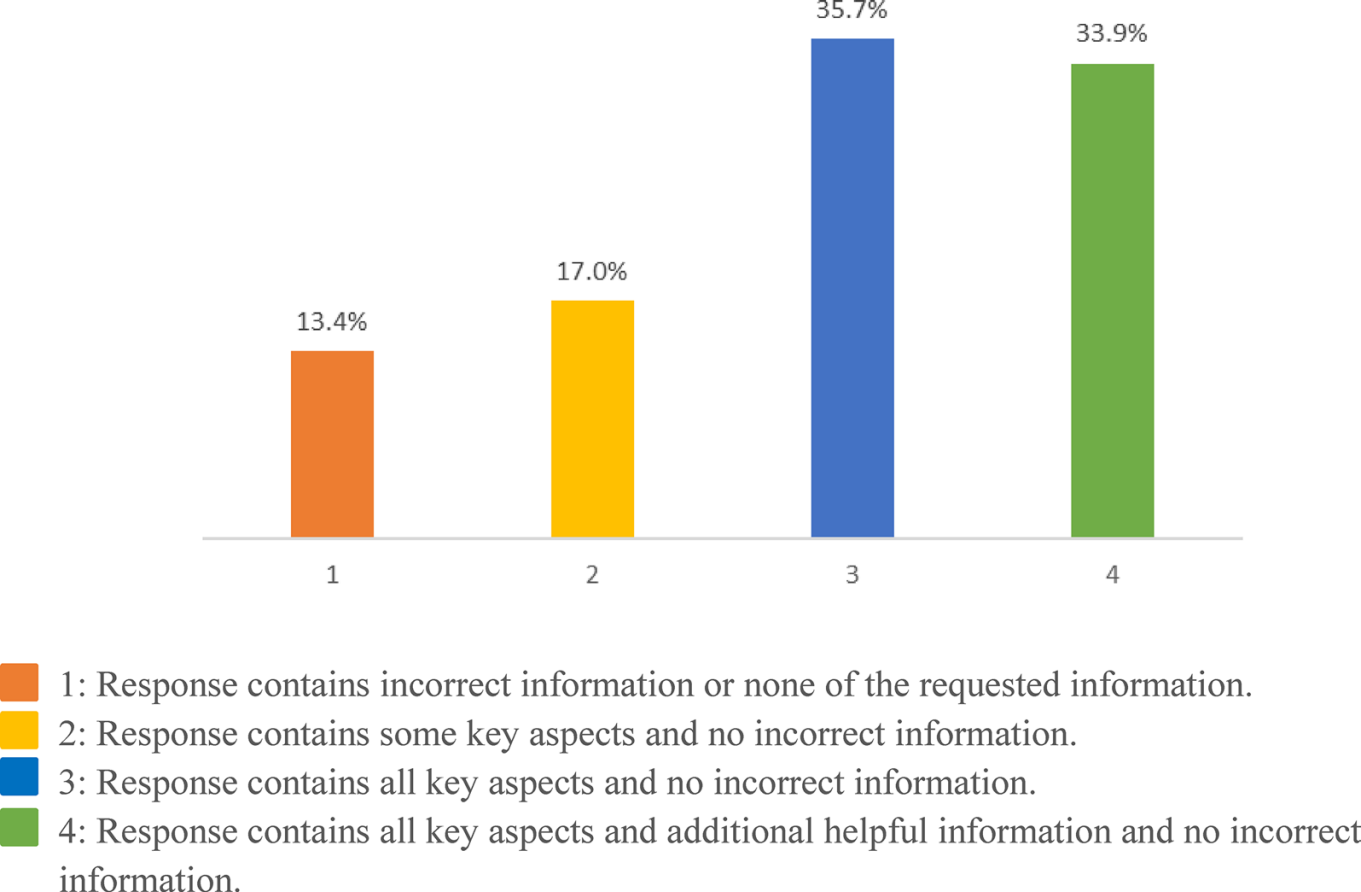
Performance in RAG tool on question answering task on guidance documents using champion LLM model (GPT-4)

Given that it was the strongest performer in the initial assessment, the performance of the GPT-4 Turbo model was further evaluated using the remaining 102 guidance documents. The retrieval process was able to find the proper source document 89.2% of the time, listing it as the first referenced source 65.2% of the time, and as the second referenced source 24.1% of the time. GPT-4 Turbo was able to generate helpful responses with useful information and no incorrect information 86.6% of the time (comprised of 33.9%, 95% CI: [25.8, 43.1] correct with additional helpful information; 35.7%, 95% CI: [27.5, 44.9] correct; and 17.0%, 95% CI: [11.1, 25.0] correct with partial information), and answers with at least some incorrect information or no relevant information 13.4% (95% CI: [8.3, 20.9]) of the time. The full results are displayed in Fig. 3. Lastly, we calculated IRR to be 0.552 for all clinical ratings in both stages of the study, indicating moderate agreement (IRR score 0.4 to 0.6) approaching substantial agreement (IRR score 0.6 to 0.8) [22].

**Fig. 4:**
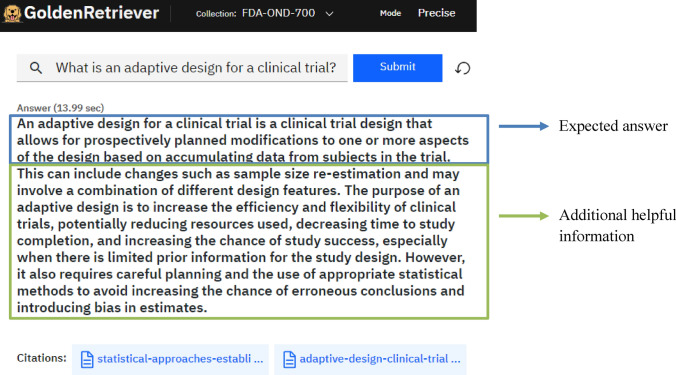
Example question and RAG tool answer scored as 4. The expected answer to the question was the definition of adaptive design for a clinical trial as seen in the first sentence. The response included additional information, such as the purpose of an adaptive design as well as an example. Thus, this answer was scored a 4

The responses most frequently included only the expected answer; however, some responses included additional information that was considered helpful. Figure 4 shows an example question and response that contained the expected answer and additional helpful information. The expected answer to the question shown in Fig. 4 was the definition of an adaptive design for a clinical trial as seen in the first sentence and as stated within the FDA guidance for industry *Statistical Approaches to Establishing Bioequivalence* [23]. The response included additional information, such as the purpose of an adaptive design, as well as an example. The clinical SMEs determined this material to be helpful, and therefore the response received a score of 4.

**Fig. 5:**
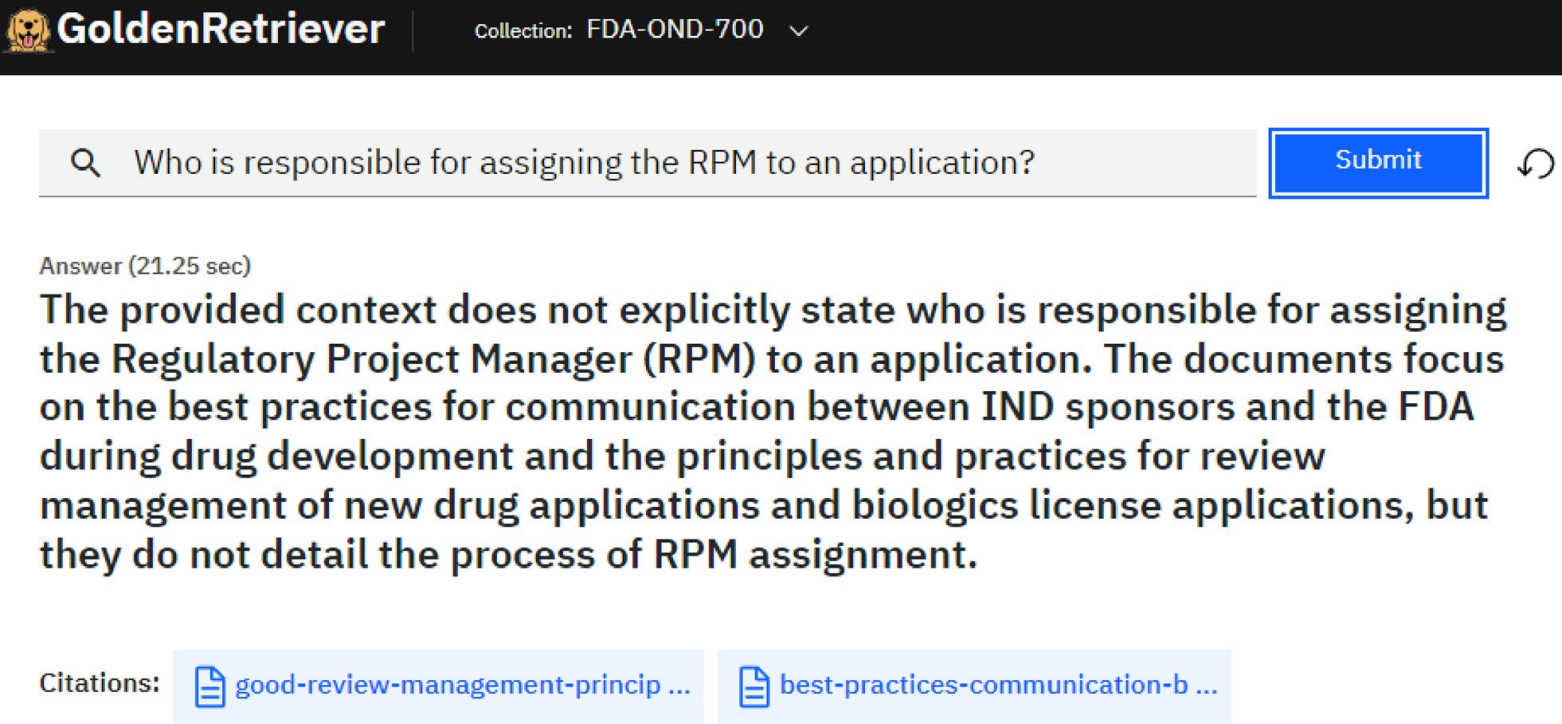
Example question and RAG tool answer scored as 1. The expected answer to the question was Chief, Project Management Staff (CPMS), as mentioned within the Guidance for Review Staff and Industry Good Review Management Principles and Practices for PDUFA Products. Thus, this answer was scored a 1

Figure 5 shows an example question and response that did not contain the expected information. The expected answer to the question was Chief, Project Management Staff (CPMS) as stated within the FDA draft guidance for review staff and industry *Good Review Management Principles and Practices for PDUFA Products* [24]. Since the response did not contain the requested information, it received a score of 1. Additionally, the guidance was not used as a reference for this response, as seen within the citations. Responses were generated from documents that were not the expected document only 10.8% of the time. For this example, the answer was provided in a table within the anticipated guidance document. Responses with an expected answer contained in a table within the guidance document most frequently received a score of 1, meaning that the information in the table was not captured in the response.

**Fig. 6:**
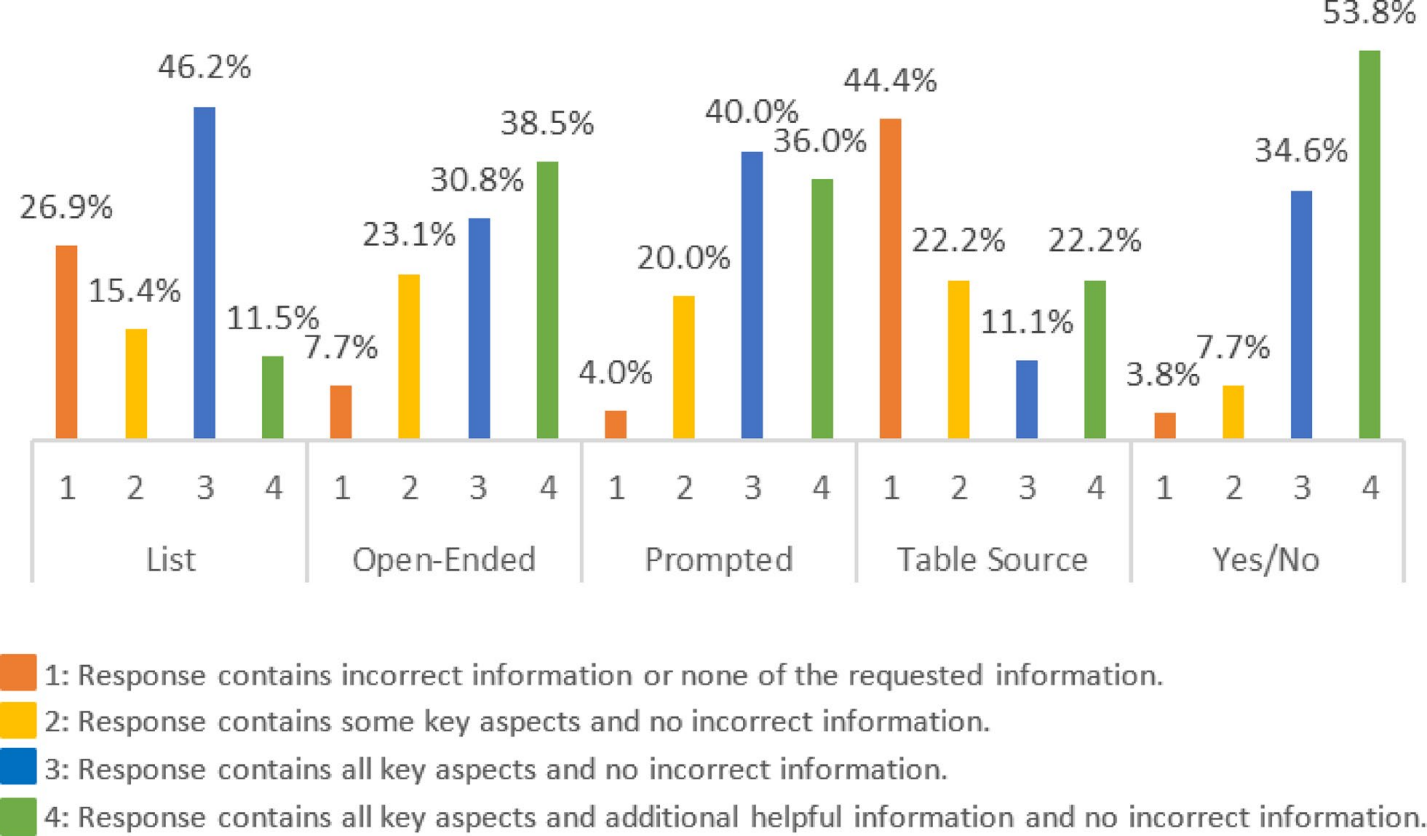
Comparison of performance of RAG tool using champion LLM (GPT-4) on question answering task on guidance documents across question types

Figure 6 and Table 3 provide the GPT-4 Turbo scoring results by question type. Responses to yes-or-no questions performed best, receiving completely correct responses (i.e., score 3 or 4) 88.4% of the time. Their responses often contained additional helpful information and received a score of 4, 53.8% of the time. Open-ended and prompted questions had similar results and received completely correct scores 69.3% and 76.0% of the time, respectively. Questions that expected a list and questions that referenced data in table format performed worst, returning responses with incorrect or no relevant information 26.9% and 44.4% of the time, respectively.

**Table 3:** Performance on different question types

Question type	Category	N	% [95% CI]
List	1	7	26.9 [13.7, 46.1]
	2	4	15.4 [61.5, 33.5]
	3	12	46.2 [28.8, 65.3]
	4	3	11.5 [4.0, 29.0]
Open-ended	1	2	7.7 [2.1, 24.1]
	2	6	23.1 [11.0, 42.1]
	3	8	30.8 [16.5, 50.0]
	4	10	38.5 [22.4, 57.5]
Prompted	1	1	4.0 [0.7, 19.5]
	2	5	20.0 [8.9, 39.1]
	3	10	40.0 [23.4, 59.3]
	4	9	36.0 [20.2, 55.5]
Table source	1	4	44.4 [18.9, 73.3]
	2	2	22.2 [6.3, 54.7]
	3	1	11.1 [2.0, 43.5]
	4	2	22.2 [6.3, 54.7]
Yes/no	1	1	03.8 [0.7, 18.9]
	2	2	7.7 [2.1, 24.1]
	3	9	34.6 [20.2, 55.5]
	4	14	53.8 [35.5, 71.2]

## Discussion

Of the five LLMs tested, GPT-4 Turbo was the highest performing, and during more extensive testing was found to generate helpful responses with no inaccuracies 86.6% of the time and to cite the source document 89.2% of the time. Unfortunately, with the remaining 13.4% of responses including some incorrect information and an additional 17% of the responses missing some element of the expected answer, this is an area of concern for the FDA. As sponsors and FDA staff rely heavily on FDA guidance documents to understand FDA’s thinking on many drug development-related issues, having 30.4% of the generative AI responses provide incomplete or incorrect information is likely not acceptable. Out of the 13.4 responses that scored a 1 (answers had incorrect or no relevant information), we did a subsequent characterization of the sources of error. Each of these question/answer pairs were broken into one of three categories: “Retrieval Process Error” (correct document not identified by the embedding model and cited by the Golden Retriever tool), “Text Extraction Error” (answer incorrect due to an error reading information from PDF tables), “LLM Poor or Confusing Wording”, and “LLM Hallucination”. Hallucinations were defined as a clearly false statement not supported by the text. This differentiated it from responses with poor or confusing wording which were also ranked as a 1 but were not clearly false. This characterization is documented in Table 4. Examples of each type of error are documented in Table s2.

**Table 4:** Characterization of error source for Golden Retriever answers that were assessed as incorrect or not related

Error category	N	%
Document text extract error	3	20.0
Wrong document retrieved	4	26.7
Wrong text section retrieved	4	26.7
LLM poor/confusing wording	3	20.0
LLM hallucination	1	6.7
Total questions	15	100.0

Additional testing on document embedding, chunking, adding more context information to the questions, and upgrading to higher performing LLMs should be conducted to assess whether higher performance of the retrieval process can be achieved. Without parameter tuning or retraining, the five LLMs that were studied do not appear to be sufficiently accurate to reliably generate responses based on FDA guidance documents without additional human review of the source document to confirm correctness. Further advancements in these models may be necessary. The models were more successful at identifying the correct source document than at generating helpful responses and could therefore be used as an enhanced document search tool if users are willing to check the source documents to confirm answers. This could mitigate the risks associated with incorrect information.

One challenge for the LLMs was with questions that used terms having different meanings depending on the context. For example, a question about “safety reports” resulted in a response that contained correct information if post-market reports were intended, but incorrect information if pre-market reports were being referenced; the tool did not clarify the distinction or request disambiguation. Of note, if we were to ingest a greater number of guidance documents, we might expect to see many more cases of terms having more than one interpretation depending on the context. In these scenarios, we should consider techniques for improving context understanding and disambiguation, such as more sophisticated prompt engineering that may incorporate domain-specific knowledge or use domain-specific data as part of the training set of an LLM to improve overall performance.

A more immediate strategy to improve access to guidance document information may involve the tool returning the relevant text within the guidance documents, as well as the generated answer, to enable users to review the document citations and confirm the answer more easily. This would be a safer approach as any mistakes by the AI would tend to be more obvious to the user, yet it would still provide users with a rapid response to their questions.

A limitation of our study is that only a small proportion of FDA guidance documents were examined, and the results may have been different if all such documents were included or if a larger sample size was used. The CIs for the first stage of the study were quite large (Table 2), making it difficult to draw firm conclusions from the results. Additionally, given that IRR only showed moderate agreement, rating answers may be an inherently difficult task or possibly the categories in our study’s scoring system were not differentiated enough from each other – especially categories 3 and 4 for correct answers. The lack of substantial clinical reviewer agreement does raise a concern that results could vary for different sets of clinical reviewers given the subjective nature of these reviews and the lack of a baseline to compare their reviews against.

Similar to many other AI tools, one of the limitations of this strategy is the continued need for human oversight which negatively impacts potential efficiency gains. In addition, future investigations should include an assessment of the time to obtain an answer manually using FDA guidance documents versus with use of the tool. However, we note that there should be a diminished need for oversight as the intended audience is FDA reviewers and other pharmaceutical industry regulatory experts who are more likely to identify substantive errors in the output compared with laypersons. In addition, if a future version of this tool were to be placed into production, we believe it would be critical to train users to confirm that the references supplied by RAG were being interpreted correctly.

An additional limitation is that the LLMs tested were all trained on general public data which may not include domain-specific information needed to understand the questions and provide accurate answers. The models could be fine-tuned on guidance documents and other domain specific text for an improvement in performance. Lastly, a final limitation of the study is that only LLMs that were available at the time of our analysis were able to be used. During time of the study, several additional, more accurate LLMs were released [25, 26]. Additional study is recommended to evaluate whether new LLM models could increase response accuracy.

## Conclusion

An application using generative AI in conjunction with a RAG architecture was able to correctly identify the relevant guidance document(s) and answer questions about the content of clinically-oriented FDA guidance documents most of the time. The RAG application returned a correct response with additional helpful information 33.9% of the time, a correct response 35.7% of the time, a response with some of the required correct information 17.0% of the time, and a response with no relevant information or any incorrect information 13.4% of the time. However, this shows that the responses contained all the relevant information only 69.6% of the time and returned an answer with incorrect information 13.4% of the time. As the information in such documents may be relied on by pharmaceutical companies and FDA staff to guide drug development decisions, the application of partial or incorrect information might have a negative impact on the drug development process. Based on these results, further research into generative AI will likely be required before it can be relied on to answer questions about the information contained in FDA guidance documents. Rephrasing questions by including additional context, testing different document embedding, chunking, or other configuration settings, or additional prompt engineering will be considered to improve the rate of fully correct and complete responses.

## Supplementary Information

Below is the link to the electronic supplementary material.

Supplementary file1 (DOCX 132 KB)

## Data Availability

No datasets were generated or analysed during the current study.
