# Supplementary material for: Semantic Search of FDA Guidance Documents Using Generative AI
Source: Ther Innov Regul Sci. 2025 Jun 14;59(5):1148–59. doi: 10.1007/s43441-025-00798-8 (PMC12446095; doi:10.1007/s43441-025-00798-8)
Supplement: Supplementary file 1 — Supplementary file1 (DOCX 132 KB) [file 43441_2025_798_MOESM1_ESM.docx]

**Supplementary Material**

**Appendix A: Prompt Template Used for Querying the LLM Using Context Extract from Guidance Documents**

Instruction ="Given a context , answer the following question from that context and only from that context." instruction_post = "Only answer from the provided context <context added here from guidance document sources."

See Tables s1 and s2.

**Table s1**

Number of questions by question type

| **Question Type** | **Count** |
| --- | --- |
| List | 24 |
| Open-Ended | 23 |
| Prompted | 23 |
| Table Source | 8 |
| Yes-or-No | 24 |

**Table s2**

Other examples of LLM answers to receive a score of 1

| **Error Reason** | **Question** | **Answer** | **Expected Answer** | **Reason** |
| --- | --- | --- | --- | --- |
| Wrong Text Section Retrieved | Provide a list of applications that can't be submitted as 505(b)(2) applications. | The provided context does not contain a list of applications that can't be submitted as 505(b)(2) applications. It mentions a section titled "IV. WHAT CAN'T BE SUBMITTED AS 505(B)(2) APPLICATIONS?" which suggests that such a list exists, but the actual content of that section is not included in the excerpts provided. Therefore, I'm unable to provide the requested list from the given context. | "· An application that is a duplicate of a listed drug and eligible for approval under section 505(j) (see 21 CFR 314.101(d)(9)); or,  · An application in which the only difference from the reference listed drug is that the extent to which the active ingredient(s) is absorbed or otherwise made available to the site of action is less than the listed drug (21 CFR 314.54(b)(1)); or,  · An application in which the only difference from the reference listed drug is that the rate at which its active ingredient(s) is absorbed or otherwise made available to the site of action is unintentionally less than that of the listed drug (21 CFR 314.54(b)(2))." | Context not provided in wrong section of text |
| LLM Poor / Confusing Wording | Generate a listing of the various types of control groups. | "The various types of control groups in clinical trials, as mentioned in the context, are:  1. Placebo Concurrent Control  2. No Treatment  3. Different Dose or Regimen of the Study Treatment  4. Different Active Treatment  5. External (Historical) Control Groups" | "1) Placebo Concurrent Control  2) No-treatment Concurrent Control  3) Dose-response Concurrent Control  4) Active (positive) Concurrent Control  5) External Control (Including historical control)" | Different Dose or Regimen of the Study Treatment is not exactly the same as different Dose or Regimen of the study treatment |
| LLM Hallucination | Provide a list of principles for assessing long term treatment of a drug for non-life-threatening conditions. | See below | See below | Although there is some overlap with the expected and LLM answer, the LLM hallucinates additional principles such as “Size of Database” |

**LLM-Generated Response:**

The principles for assessing the clinical safety of drugs intended for long-term treatment of non-life-threatening conditions, as outlined in the context provided, include:

1. **Safety Evaluation Duration**: The safety evaluation during clinical drug development should characterize and quantify the safety profile of a drug over a reasonable duration of time consistent with the intended long-term use of the drug.

2. **Size of Data Base**: The size of the data base necessary to achieve safety evaluation goals should be determined by considering the duration of drug exposure and its relationship to both time and magnitude of occurrence of adverse events.

3. **Characterization of Adverse Drug Events (ADEs)**: Short-term event rates should be well characterized, and events where the rate of occurrence changes over a longer period may need to be characterized depending on their severity and importance to the risk-benefit assessment of the drug.

4. **Design of Clinical Studies**: The design of clinical studies can significantly influence the ability to make causality judgments about the relationships between the drug and adverse events. Different types of control groups (placebo, positive, or none) impact the ability to assess causality.

5. **Harmonized Regulatory Standard**: A harmonized regulatory standard is valuable for the extent and duration of treatment needed to provide the safety data base for drugs intended for long-term treatment of non-life-threatening conditions.

6. **Regulatory Standards Based on Experience**: Regulatory standards for the safety evaluation of drugs should be based on previous experience with ADEs, statistical considerations, and practical considerations.

7. **Incomplete Information on ADEs**: Information about the occurrence of ADEs in relation to duration of treatment for different drug classes is incomplete, and further investigations to obtain this information would be useful.

8. **Frequency and Timing of ADEs**: Most ADEs first occur and are most frequent within the first few months of drug treatment. The number of patients treated for 6 months should be adequate to characterize the pattern of ADEs over time.

9. **Cohort Size**: Usually, 300 to 600 patients should be adequate to observe whether more frequently occurring events increase or decrease over time as well as to observe delayed events of reasonable frequency.

10. **Longer Duration for Some Patients**: Some patients should be treated with the drug for 12 months to detect ADEs that may increase in frequency or severity with time or that may occur only after more than 6 months of treatment.

11. **Specific Number of Patients for One-Year Follow-Up**: 100 patients exposed for a minimum of one year is considered acceptable to include as part of the safety data base, providing reasonable assurance that the true cumulative one-year incidence of serious ADEs is no greater than 3%.

These principles are intended to guide the safety evaluation of drugs for long-term use in treating non-life-threatening conditions, ensuring that the safety profile is adequately assessed and characterized.

**Expected Answer:**

1. A harmonized regulatory standard is of value for the extent and duration of treatment needed to provide the safety data base for drugs intended for long-term treatment of non-life-threatening conditions. Although this standard covers many indications and drug classes, there are exceptions.

2. Regulatory standards for the safety evaluation of drugs should be based on previous experience with the occurrence and detection of adverse drug events (ADEs), statistical considerations of the probability of detecting specified frequencies of ADEs, and practical considerations.

3. Information about the occurrence of ADEs in relation to duration of treatment for different drug classes is incomplete, and further investigations to obtain this information would be useful.

4. Available information suggests that most ADEs first occur, and are most frequent, within the first few months of drug treatment. The number of patients treated for 6 months at dosage levels intended for clinical use, should be adequate to characterize the pattern of ADEs over time. To achieve this objective the cohort of exposed subjects should be large enough to observe whether more frequently occurring events increase or decrease over time as well as to observe delayed events of reasonable frequency (e.g., in the general range of 0.5%-5%). Usually 300 to 600 patients should be adequate.

5. There is concern that, although they are likely to be uncommon, some ADEs may increase in frequency or severity with time or that some serious ADEs may occur only after drug treatment for more than 6 months. Therefore, some patients should be treated with the drug for 12 months. In the absence of more information about the relationship of ADEs to treatment duration, selection of a specific number of patients to be followed for 1 year is to a large extent a judgement based on the probability of detecting a given ADE frequency level and practical considerations. 100 patients exposed for a minimum of one-year is considered to be acceptable to include as part of the safety data base. The data should come from prospective studies appropriately designed to provide at least one year exposure at dosage levels intended for clinical use. When no serious ADE is observed in a one-year exposure period this number of patients can provide reasonable assurance that the true cumulative one year incidence is no greater than 3%.

6. It is anticipated that the total number of individuals treated with the investigational drug, including short-term exposure, will be about 1500. Japan currently accepts 500 to 1500 patients; the potential for a smaller number of patients is due to the postmarketing surveillance requirement, the actual number for a specific drug being determined by the information available on the drug and drug class.

7. There are a number of circumstances where the harmonized general standards for the clinical safety evaluation may not be applicable. Reasons for, and examples of, these exceptions are listed below. It is expected that additional examples may arise. It should also be recognized that the clinical data base required for efficacy testing may be occasionally larger or may require longer patient observation than that required by this guideline.

8. Filing for approval will usually be possible based on the data from patients treated through 6 months. Data on patients treated through 12 months should be submitted as soon as available and prior to approval in the United States and Japan but may be submitted after approval in the European Union. In the United States, the initial submission for those drugs designated as priority drugs must include the 12 months patient data.

**Figure s1**

Overview of Golden Retriever RAG tool user interface


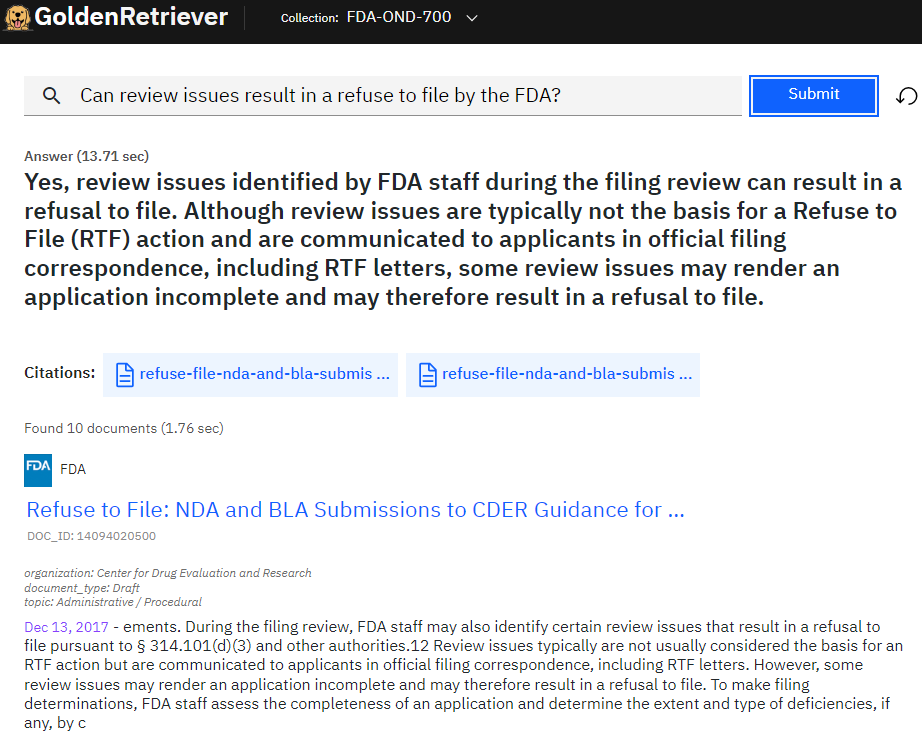


Question field

Generated response

List of citations

Citation document

Citation document metadata
